# Reproducing American Sign Language sentences: cognitive scaffolding in working memory

**DOI:** 10.3389/fpsyg.2014.00859

**Published:** 2014-08-08

**Authors:** Ted Supalla, Peter C. Hauser, Daphne Bavelier

**Affiliations:** ^1^Sign Language Research Lab, Department of Neurology, Center for Brain Plasticity and Recovery, Georgetown UniversityWashington, DC, USA; ^2^Department of American Sign Language and Interpreting Education, Deaf Studies Laboratory, National Technical Institute for the Deaf, Rochester Institute of TechnologyRochester, NY, USA; ^3^Department of Psychology and Education Sciences, University of GenevaGeneva, Switzerland; ^4^Department of Brain and Cognitive Sciences, University of RochesterRochester, NY, USA

**Keywords:** American Sign Language, working memory, error analysis, verbatim recall, native signers, reproduction error, error type

## Abstract

The American Sign Language Sentence Reproduction Test (ASL-SRT) requires the precise reproduction of a series of ASL sentences increasing in complexity and length. Error analyses of such tasks provides insight into working memory and scaffolding processes. Data was collected from three groups expected to differ in fluency: deaf children, deaf adults and hearing adults, all users of ASL. Quantitative (correct/incorrect recall) and qualitative error analyses were performed. Percent correct on the reproduction task supports its sensitivity to fluency as test performance clearly differed across the three groups studied. A linguistic analysis of errors further documented differing strategies and bias across groups. Subjects' recall projected the affordance and constraints of deep linguistic representations to differing degrees, with subjects resorting to alternate processing strategies when they failed to recall the sentence correctly. A qualitative error analysis allows us to capture generalizations about the relationship between *error pattern* and the cognitive scaffolding, which governs the sentence reproduction process. Highly fluent signers and less-fluent signers share common chokepoints on particular words in sentences. However, they diverge in heuristic strategy. Fluent signers, when they make an error, tend to preserve semantic details while altering morpho-syntactic domains. They produce syntactically correct sentences with equivalent meaning to the to-be-reproduced one, but these are not verbatim reproductions of the original sentence. In contrast, less-fluent signers tend to use a more linear strategy, preserving lexical status and word ordering while omitting local inflections, and occasionally resorting to visuo-motoric imitation. Thus, whereas fluent signers readily use top-down scaffolding in their working memory, less fluent signers fail to do so. Implications for current models of working memory across spoken and signed modalities are considered.

## Introduction

Current literature in psycholinguistics and cognitive science has deepened our understanding of the nature of short term memory (STM), but much work remains in the description and modeling of working memory, particularly for understanding the impact of modality on language processing. Working memory is generally considered to be a scaffolding for cognitive functions required to accomplish a task (Baddeley, 1995). However, debate goes on as to whether the layers of linguistic processing are modular or interactive (Fodor, 1983; Just and Carpenter, 1992) and whether STM is separable from working memory (Baddeley and Hitch, 2007). Research into the nature of STM in a signed language so far reveals that STM capacity for individual signs is not identical to the processes and capacity used in the recall of spoken words (Boutla et al., 2004; Bavelier et al., 2006). One may ask what this implies for the working memory of signers in processing sentences. One way to address this question is to examine the way signers use working memory to process and retain ASL sentences. To pursue this line of research, we have examined ASL sentence reproduction, particularly the effect of bottleneck conditions on this task. We hypothesized that there are similar kinds of processes and constraints on working memory for processing ASL sentences and spoken sentences. Furthermore, we hypothesized that during cognitive and linguistic encoding and production, fluent signers make use of linguistic scaffolding and parsing options that are not available to signers with lower levels of competence. An error analysis of signers across a range of fluency levels supports these hypotheses, with generalizations from the data consistent with current models of language processing, supporting processes of grammatically constrained regeneration of conceptual content (Potter and Lombardi, 1990) and showing the effects of effortful “explicit” processing at the lexical level (Ronnberg et al., 2008) in less fluent signers.

Psycholinguistic investigations of both signed and spoken language have shown that performance on many types of working memory tasks interacts significantly with language fluency and age of acquisition (Newport and Meier, 1985; Newport, 1990). In a seminal study of ASL sentence shadowing and recall, Mayberry and Fischer (1989) showed error patterns which point to different types of processing by native and late learners of the language. Native signers' errors were predominantly lexical substitutions that had a semantic relationship to the target sign and were unrelated to its phonological form. In contrast, later-learning signers' errors were predominantly those with a formational or phonological relationship to the target, but not to its meaning^1^. While subjects from both groups made both types of errors, they produced these errors in strikingly different proportions. Morford (2002) has proposed that early language exposure enables automaticity of phonological processing, one factor which may account for the difference in relative proportion of error types. Thus, the Mayberry and Fischer (1989) error pattern data can be framed as an interaction between automatic linguistic processing and conceptual regeneration for sentence shadowing. In these terms, native signers make semantic errors consistent with automatic processing, storing the content in conceptual terms, whereas late learners and less fluent signers might be seen to make more superficial errors because of their more limited abilities to process through the phonology to achieve a deeper representation of the sentence. In both the Mayberry and Fischer study and the study reported on in this paper, the detailed examination of error patterns in response to controlled target stimuli by groups of signers differing in aspects of hearing status, age and signing background reveals processing strategies and details which can illuminate our understanding of models of working memory.

The methodology of our study on working memory differs from Mayberry and Fischer's (1989) shadowing task in several ways. First, we ran subjects from a variety of linguistic backgrounds and pooled subjects in specific population groups to examine error distribution. Second, whereas Mayberry and Fischer used short, easily remembered sentences, our stimuli ranged from short, easily recalled sentences to longer and/or more complex utterances. This difference in stimuli and task led to a greater number and variety of error types for the American Sign Language Sentence Reproduction Test (ASL-SRT) data. In particular, while the Mayberry and Fischer data analyses categorized only semantic and phonological substitution types, our data analysis also included syntactic substitutions such as changes in sign order and morphological alternations. This richness in turn has allowed us to provide a more detailed analysis of the various constraints and processes at play during working memory for linguistic materials, and contributes to the development of a processing model that highlights a number of new and key features in linguistic working memory.

The ASL-SRT assesses ASL language proficiency by asking subjects to repeat verbatim a 20-item series of ASL sentences. Over the course of the test, the sentences increase in length, number of propositions, and morphological complexity (Hauser et al., 2008). In the present study, we examined in more depth the data of selected adult and young Deaf subjects from this study, as well as incorporating data from an additional pool of hearing subjects who also took this test, but whose data was not included in Hauser et al. (2008). All subjects had deaf parents who used ASL in the home. While all subjects were exposed to American Sign Language in homes with Deaf parents, various other factors affect their ASL proficiency at the time of testing. Within the sign language community, there is variation in the age at which signers are first exposed to ASL and in the input they receive to the language. Moreover, there is also variation in fluency even among native signers. Fluency increases with age (young children as compared with older children and adults) and also varies according to the extent of immersion and use of the language. For example, Deaf native signers often differ from Hearing native signers in whether ASL is their dominant language and how much they use the language in daily life. As a result of such differences, signers may or may not demonstrate fluency and a high level of proficiency in the sign language to which they are exposed. Including both the Deaf of Deaf Adults (DDA) (those raised by Deaf parents) and Deaf of Deaf Youths (DDY) groups allows us to examine the effect of age upon reproduction skills, while including the Hearing of Deaf Adults (HDA) group allows us to contrast the performance of variously fluent hearing signers with the Deaf groups while keeping home language backgrounds constant. In this way, we avoided confounding hearing status with L2 language issues. Interestingly, we found little evidence of intrusion of English grammar in the pool of 75 signers. One DDY added English features occasionally. For example, he replaced the ASL sign HAVE-TO with an English-based sequence of signs glossed as HAVE TO. The variation in fluency among the hearing offspring of Deaf parents is similar to the range from highly fluent to semi-speaker in children from minority or immigrant ethnic group families where parents continue using their native language at home. Possible factors affecting their fluency are the number of deaf siblings, if any, and birth order of the HDA subject.

The reproduction accuracy of all signers was examined as well as the nature of their response. All subjects took a previous version of the test with 39-items, but we examined data only from responses to the 20 test items included in the current version of the test. The determining factors for eliminating test sentences were the measured redundancy of some test items that showed a similar level of complexity, and the potential for inconsistencies from dialectal variation for particular items. The analysis of this sample establishes the effectiveness of the reproduction task as a tool for measuring fluency in a sign language, showing that the test is indeed sensitive to the differences in linguistic structure of signing among signers of varying ages and fluency. Furthermore, the analysis confirms that native signing raters can reliably differentiate the accuracy of reproduction across groups whom we would expect to differ in fluency with more technical linguistic assessments of grammatical structure.

In this article, we first provide the definitive description of the ASL-SRT. We then discuss the quantitative analyses performed on the three groups of native signing subjects who took the test. We also outline the method and results from qualitative analyses of the ASL-SRT responses from this same pool of 75 participants. The data reveal that signers' error types differ according to individuals' relative level of competence, as measured by their reproduction accuracy. The stimuli and task are sensitive to the subjects' differing levels of exposure and use of ASL, with performance analyses showing that signers varied in success in reproducing a target form, even in a short, single-clause sentences. Moreover, Deaf and Hearing signers who obtained higher reproduction accuracy scores made different sorts of errors than weak signers. Among less fluent signers, responses often include ungrammatical sign forms and/or sentences. Furthermore, errors are less predictable than those of more fluent signers as sentences increase in complexity. In more fluent signers, complex sentence targets trigger specific processing difficulties and predictable types of errors. The escalating demands of the reproduction task also result in clusters of various types of errors, which are useful for teasing out processing at the interface between the layers of processing and specific phrasal domains.

We developed the American Sign Language Sentence Reproduction Task (ASL-SRT) with the goal of establishing a standardized instrument that could be used across age and ability level to assess proficiency and fluency of signers. In the responses of subjects, we see differences in overall reproduction accuracy as a reflection of signers' various levels of sign language exposure, use and resultant fluency. In addition, we see differences in the types of errors made by signers of different fluency levels and backgrounds. From the perspective of a cognitive scientist, the precisely controlled data from the ASL-SRT provide an opportunity to examine the way signers use working memory to process and reproduce sentences.

The error patterns across variably fluent groups have implications for current models of working memory across spoken and signed modalities. That is, the conventional model of serial processing for non-sentence material can be replaced by a hierarchical model for working memory with parallel processing capabilities, a top-down scaffolding mechanism that assists sentence reproduction. The error analyses presented here portray a psychologically real representation of this model via performance generalizations. In turn, this model accounts for how the cognitive system executes heuristic operations across domains and levels in both a serial and parallel fashion, thus making it possible to explain clusters of multiple errors in the ASL-SRT task.

## Materials and methods

### The american sign language sentence reproduction task (ASL-SRT)

The ASL-SRT was developed for sign language by adapting the approach used in the spoken-language Test of Adolescent Language 3 (TOAL3), Speaking/Grammar subtest (Hammill et al., 1994). Like the TOAL3, this test presents sentences in gradually increasing complexity and asks the subject to repeat the sentence exactly. The 20^2^ test items are graduated in difficulty, increasing in length of sentence, complexity of morphology, and number of propositions; Table 1 lists word span, syntactic complexity, and content for each item. The first 10 test items are single clause sentences with a variety of argument-predicate relations, as shown in the top half of Table 1. In contrast, Items 11–20 contain multiple clauses with various types of relations among constituents.

**Table 1:** **Word span and syntactic complexity of ASL-SRT items, with sentence content and inflections**.

**Item**	**Word span**	**Syntactic complexity**	**Sentence content and inflections**
1	5	Transitive predication	INDEX-first FINISH BUY OLD HOUSE
2	3	Adjectival predication	THAT-i TREE TALL
3	4	Transitive predication	INDEX-i FINISH FIND KEY
4	6	Adjectival predication	MY LAST VACATION SEVEN YEARS AGO
5	4	Adjectival predication	THAT MAN NICE SWEET
6	4	Transitive predication	INDEX-i NOT LIKE INDEX-j
7	4	Adjectival predication	SUNDAY NEWSPAPER TEND CL: thickness-on-surface
8	4	Adjectival predication	MY DAUGHTER SELF-i AGE-THREE
9	4	Intransitive action	MY DOG CONTINUE+rep BARK
10	4	Adjectival predication	WOMAN SELF-i COMPETENT MATH
11	7	Copular object NP, adjectival predication	WASHINGTON #DC HAVE MANY GOVERNMENT BUILDING, CL: huge-object-alternating-ijk
12	4	Adverbial predication, intransitive action	INDEX-first DRIVE FIVE-HOUR, ARRIVE WORN-OUT
13	7	Conditional clause with transitive predication, consequence clause with adverbial predication	IF INDEX-i NOT BELIEVE INDEX-self, THAT FINE
14	4	Conjunction of intransitive action and locative predication	MOTORCYCLE CL: vehicle-slide-off-ground, HIT TREE
15	6	Locative predication, Transitive predication, Locative predication	WOMAN RIDE-horse HORSE, SEE-i FENCE, CL:jump-over-fence-i
16	6	Locative predication, intransitive action	THREE-OF-US GO-i-rep GRANDMOTHER HOUSE, HELP CLEAN-UP-arc-i
17	6	Locomotion, Locative predication, POV predication	INDEX-first LIKE GO BIKE PATH CL: trees-go-by
18	7	Transitive predication, Object complement, adjectival predication	#DAVID GO WATCH-i MAN LECTURE, CL: in-back-of-audience FULL
19	9	Transitive predication, transitive predication	SCIENCE TEACHER DISTRIBUTE TEST, INDEX-arc STUDENT HAVE-TO NAME+rep-on-list STAR
20	7	Locative predication, transitive predication	ONE LITTLE GIRL GO OUT, FLOWER CL: pick-up/ put-in-basket+rep-arc

The test is administered on a laptop computer. Subjects view a video of a woman who serves as both an instructor and a model producing the set of practice and test sentence items. She instructs subjects to copy the model's exact signing, stressing the need for verbatim response. This instruction is followed by three practice sentences with subjects responding. In the review of the practice items, the instructor compares two versions of the signs YESTERDAY and DARK for which she used one version in the practice session and presents the common alternate form, showing movement and handshape variants and instructing subjects to copy the exact parameters used by the signing model for each sentence. The test session follows and is self-paced without a time limit for response: subjects view each sentence only once, but they then have unlimited time to make their response. Thus, subjects may self-correct or repeat a response before moving on the next sentence by pressing a key. On average, it takes a subject 10 minutes to complete the test.

The responses were video-recorded and the rating took place later. In the case of repeated responses, raters were instructed to use the last response for rating purposes. In the absence of any response before moving on the next sentence, raters were instructed to mark the sentence item as a failure. On average, a complete rating of a subject's 20-response set takes 20 minutes.

### Rater training

One compelling reason for pursuing this method for measuring ASL proficiency is that the test is easy to administer and can be scored with robust inter- and intra-rater reliability by native signers, even those without a linguistics background, following minimal training. Both rater training and scoring takes place with raters blind to the hearing status of the subjects. The training materials consist of a DVD, which includes training and practice videos in ASL, and downloadable rating sheets, scoring symbol keys and guidelines for the scoring of test sentences. Raters complete blind practice with sample training subjects drawn from a wide range of signing fluency, from novice to highly fluent. This enables them to develop metalinguistic skills for assessing a range of performance levels and familiarizes them with acceptable and unacceptable variation in sentence reproduction. It is not clear whether non-native signers can be trained to achieve high accuracy in rating; we have focused on using native signers for this role, since it may be difficult for non-native signers to notice some of the errors that they might well make themselves.

This rater-training protocol provides an introduction to the overall accurate reproduction aim of the ASL-SRT and to the two aspects of rating: scoring each item as correctly or incorrectly reproduced, and noting the type of error in the case of incorrect reproduction at various levels. Raters also are introduced to the internal structure of the test, with sentences arranged in increasing levels of length and complexity. The rater-training tutorial proceeds through the following process: raters first build skills and familiarity with judging reproduction accuracy with the basic test sentences 1–10. They then proceed on to accuracy and error type notation for the more complex sentences 11–15 and 16–20, following a mid-point review of their skills with additional instruction. In general, the rater training takes about 3 days to complete.

### Subject pool

For the analysis described here, subjects were recruited from deaf college programs and from summer camp programs for deaf children and hearing children with deaf parents. The subject pool was comprised of signers from three groups: Native Deaf adult signers (DDA), ages 15–30 (*N* = 25); native Deaf young signers (DDY) ages 10–14 (*N* = 25); and native Hearing adult signers (HDA), often known as Children of Deaf Adults, or CODAs, ages 15–30 (*N* = 25). The protocol was approved by the Institutional Review Boards of the University of Rochester and the Rochester Institute of Technology, and all subjects gave informed consent.

## Results

Seventy-five participants took the ASL-SRT and five trained raters rated the participants' sign reproductions independently. The inter-rater reliability was high and correlation coefficients ranged from 0.86 to 0.92.

For each participant, ASL-SRT performance was indexed by two different measures. First, reproduction was scored as correct or incorrect based on an all-or-none scheme whereby any error in reproduction would lead to a zero score. Second, more detailed analyses were carried out classifying errors by type and recording the frequency of each type of error within and between participant groups.

### Overall response accuracy analysis

**Figure 1:**
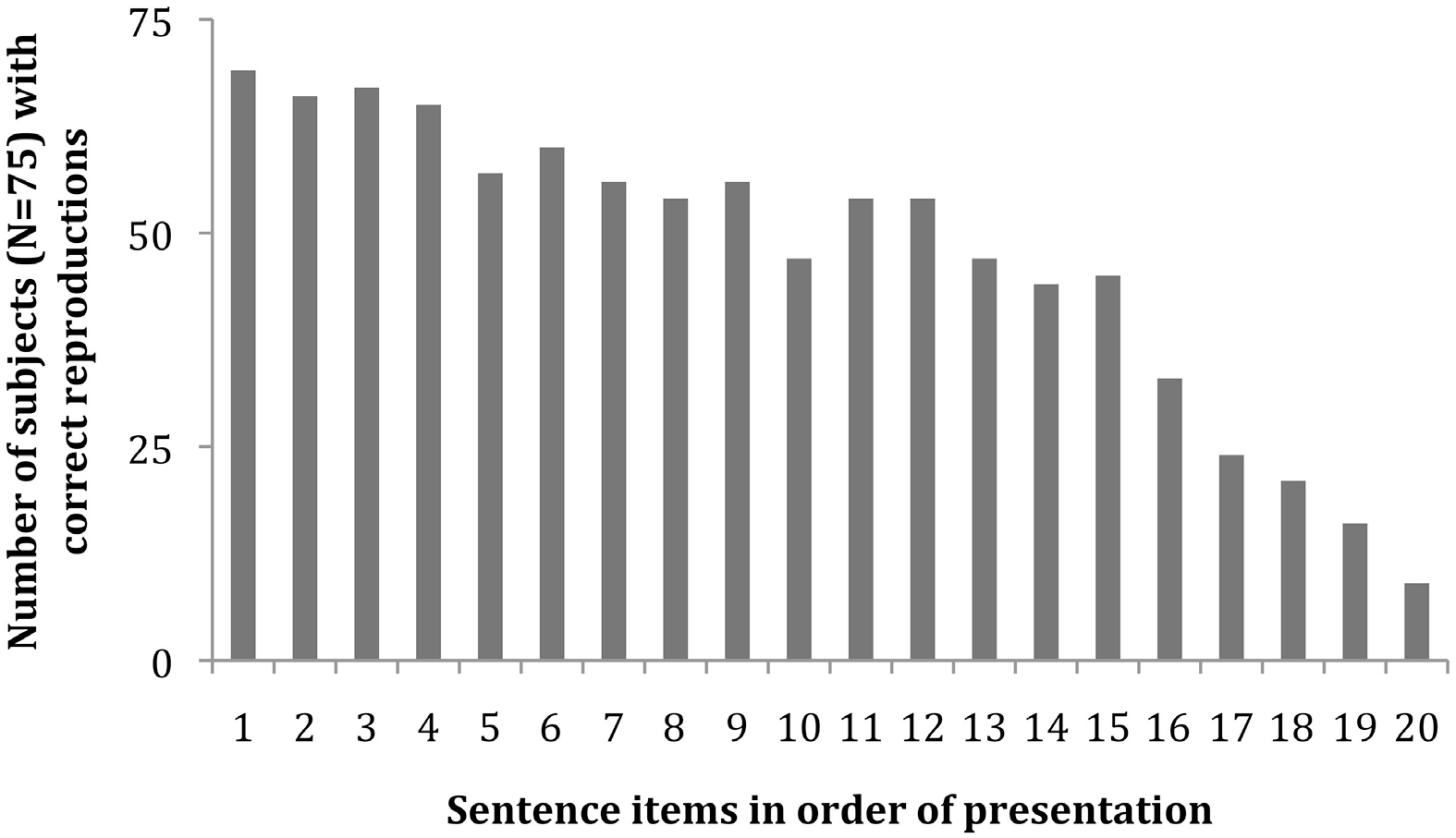
**Histogram of participants with correct reproduction for each of the 20 sentences in the ASL-SRT task (*N* = 75 subjects)**.

Figure 1 shows the number of subjects in the 75-subject pool who accurately reproduced each of the test's 20 sentences. The slope indicates an overall increasing difficulty in sentence reproduction, reflecting the increasing complexity of ASL grammatical structure from sentence 1 to 20.

**Figure 2:**
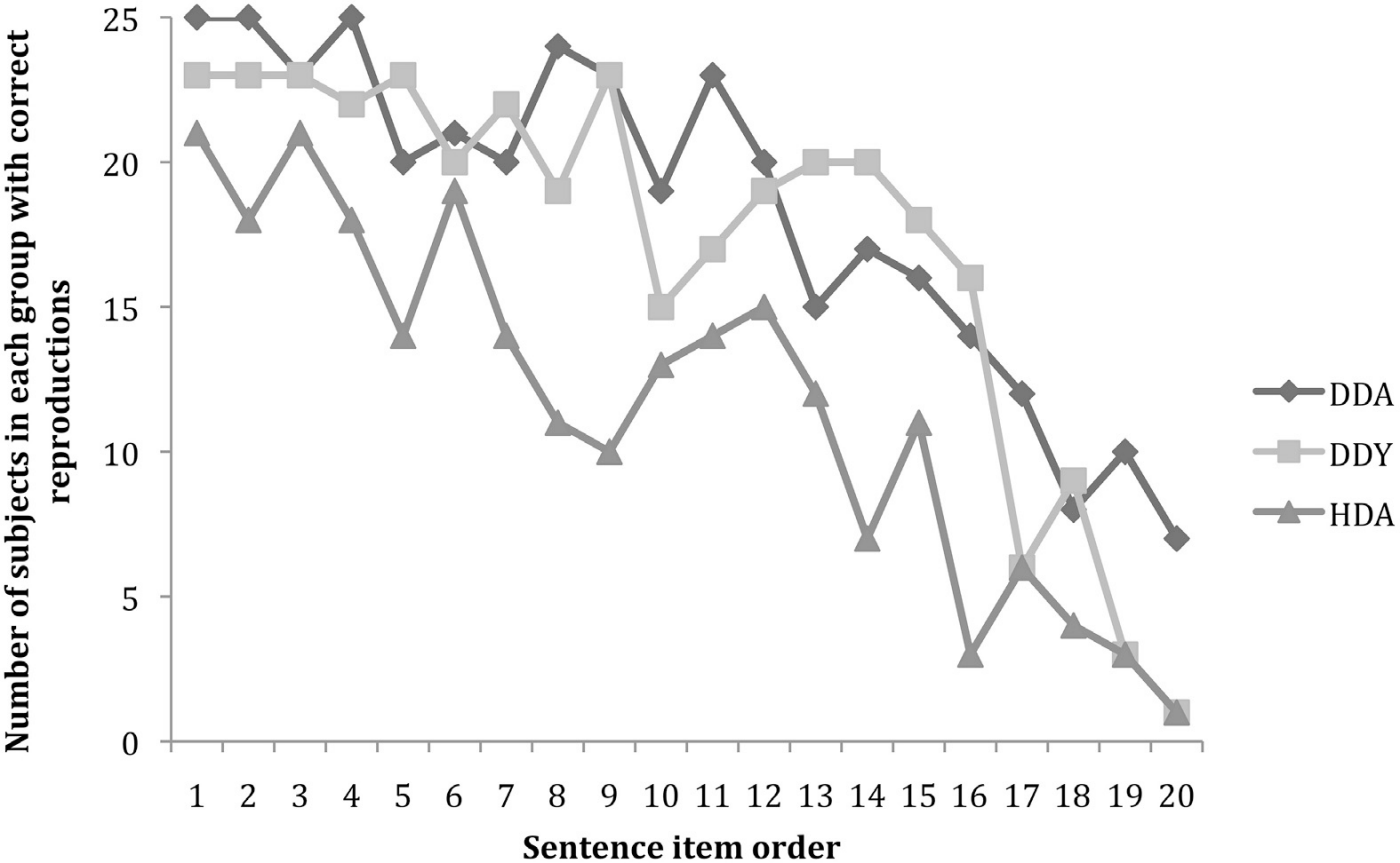
**Number of participants per group (maximum *N* = 25) with correct sentence reproduction as a function of sentence complexity ordered from easiest (sentence 1) to hardest (sentence 20)**.

The overall trend for each group for performance across the 20 sentences is shown in Figure 2. Grouping the subjects by similar home backgrounds but differing age and hearing status can ultimately help us to tease out which experiential factors may be responsible for the various fluency levels shown by the subjects.

An ANOVA was conducted with Group (DDA, DDY, HDA) as the between group factor and number of correct sentences reproduced as the dependent variable. A significant group difference was found, *F*_(2, 72)_ = 16.001, *p* < 0.001; partial eta squared = 0.308. *Post-hoc* analyses revealed no significant difference between the two deaf groups, DDA (*M* = 14.7; *SD* = 2.8) and DDY (*M* = 13.7; *SD* = 3.2). However, young and adult deaf signers were able to reproduce more ASL-SRT sentences than HDA (*M* = 9.4; *SD* = 4.3).

### Error type analyses

The remaining analyses in this paper are based on the error ratings of the first author of this article who served as a rater for this 75-subject pool and the rating trainer. The analysis of sentence reproduction failures by the 75 subjects provides useful information on the trends for particular error types along the 20-sentence range of incremental complexity. For each incorrect reproduction response, the first three errors identified in the sequence of signs for each sentence were collected for quantitative analyses here (See Table 2).

**Table 2:** **Distribution of errors across 1500 responses**.

**Subject group**	**# test items**	**Successful reproduction**	**Failure**	**With 1 error**	**With 2 errors**	**With 3 errors or more**
DDA (*n* = 25)	500	361	139	77	34	28
DDY (*n* = 25)	500	348	152	74	49	29
HDA (*n* = 25)	500	247	253	121	80	52
*N* = 75	1500	956	544	272	163	109

Analysis of the first 3 errors as a methodological protocol captured the vast majority of errors and was well within and often beyond the average number of errors that signers made in a single sentence. At any given word location within a sentence, multiple errors were noted as well. The complete range of error types is explained and exemplified in Table 3.

**Table 3:** **Classification of reproduction errors**.

**Error type and description**	**Example**
**OMISSION AS ONE TYPE OF ERROR**	
Target sign is omitted	Stimulus: MOTORCYLE CL: vehicle-spin HIT TREE
	Response: MOTORCYCLE HIT TREE
**MORPHOLOGICAL TYPE OF ERROR**	
Bound inflectional morphology is replaced, resulting in simplified sign (morphological omission)	Stimulus: MY DOG CONTINUE+++ BARK
	Response: MY DOG CONTINUE BARK (no reduplication for continuous aspect)
Re-interpretation of classifier structure such that response has similar form but different meaning	Stimulus: INDEX-first LIKE GO BIKE PATH CL: trees-go-by
	Response: INDEX-first LIKE GO BIKE PATH CL: wind-in-air
Merge two signs into one form	Stimulus: MOTORCYLE CL: vehicle-spin HIT TREE
	Response: MOTORCYCLE CL: vehicle-spin CL: vehicle-hit-tree
**LEXICAL TYPE OF ERROR**	
The target sign is replaced by a different lexical form (lexical substitution)	Stimulus: MOTORCYCLE CL: vehicle-spin HIT TREE
	Response: MAN CL: vehicle-spin HIT TREE
Sign not present in the stimulus item is added (lexical commission)	Stimulus: MOTORCYCLE CL: vehicle-spin HIT TREE
	Response: MOTORCYCLE CL: vehicle-spin HIT TREE CL: person-fall-from-vehicle
**PHONOLOGICAL TYPE OF ERROR**	
Response sign is misarticulated in form, thus recognized as different from the target sign (misarticulation)	Stimulus: MOTORCYCLE SPIN(base hand palm-down) HIT TREE Subject Error: MOTORCYCLE SPIN(base hand palm-up) HIT TREE
Sign is replaced with one that was morphologically/lexically related (morpho-phonemic substitution)	Stimulus: MY DOG CONTINUE+++ BARK
	Response: MY DOG CONTINUE+++ BITE
**SYNTACTIC TYPE OF ERROR**	
Sequence of signs is reordered (word displacement)	Stimulus: INDEX-first LIKE GO BIKE PATH CL: trees-go-by
	Response: INDEX-first LIKE GO CL: trees-go-by BIKE PATH
A sign is repeated at a different location in the sentence	Stimulus: INDEX-first LIKE GO BIKE PATH CL: trees-go-by
	Response: INDEX-first LIKE GO BIKE PATH CL: trees-go-by BIKE
**OTHER RESPONSE ERROR TYPES**	
Rough approximation of form and movement of target sign (visuo-motoric mimicry)	Overall response has a meaningless non-sign approximating the phonology of the target sign at a particular sentence location
Miscomprehension: Response indicates that subject does not understand concepts in stimulus	Stimulus: MOTORCYCLE SPIN HIT TREE
	Response: MOTORCYCLE RIDE SEE TREE

The reproduction error types listed in Table 3 do not include linguistic deviations reflecting factors such as dialect, age-related experiential differences, and permissible phonological variations in ASL. Rater tolerance to variation was resolved with tutorials where raters were exposed to 10 models including a mixture of novice, semi-fluent, and fluent signers. Rather, error types were incorrect reproductions and not merely pronunciation or accent differences.

In lexical and morpho-phonemic substitutions and morphological merging errors, subjects substituted a different sign than the one in the stimulus model in a given sentence location. Such data allow us to flesh out and further subdivide the notion of “semantic error” as described in Mayberry and Fischer (1989). In lexical and syntactic errors, we see a further distinction between errors preserving semantic content at the lexical level (synonyms) vs. errors preserving semantic content through grammatical alternations, affecting the morpho-syntactic structure of the entire sentence. In cases of multiple alternations or commissions in a given sentence location, each deviation is counted as a separate error.

**Figure 3:**
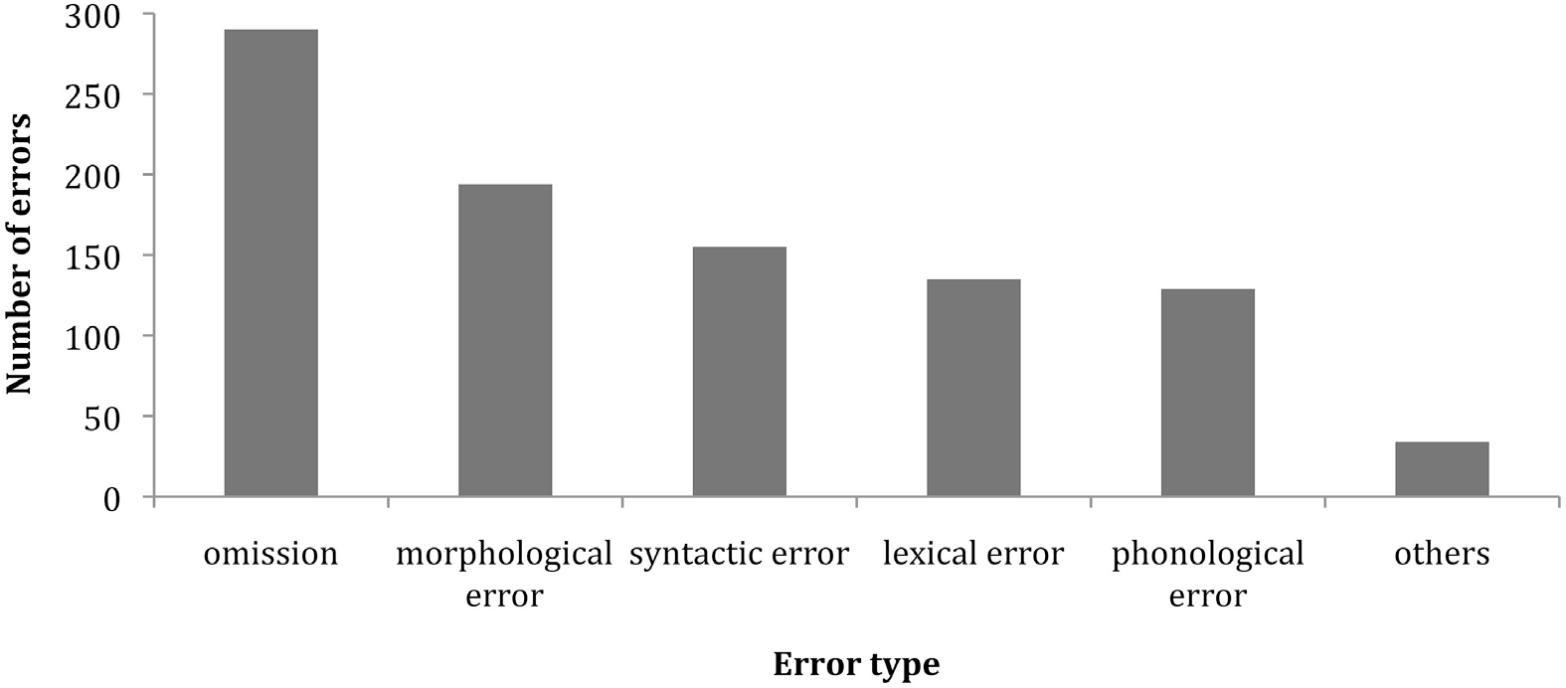
**Incidence and relative proportions of error types across 75 subjects**.

In the next section we turn to the main factor affecting reproduction success and error type: the relative fluency of the signer. To begin, Figure 3 shows the number of occurrences of six separate error types in the pooled 75-subject response data for 20 sentences. Each of the first 3 errors was included in the count, including duplicate types of errors within a particular subject response.

Word omission is the most frequently occurring type of error, with a higher incidence than the remaining error types: morphological, syntactic, lexical, and phonological. There is no statistically significant difference among the latter four error types in overall frequency of occurrence.

#### Error types as a function of hearing status and age

**Figure 4:**
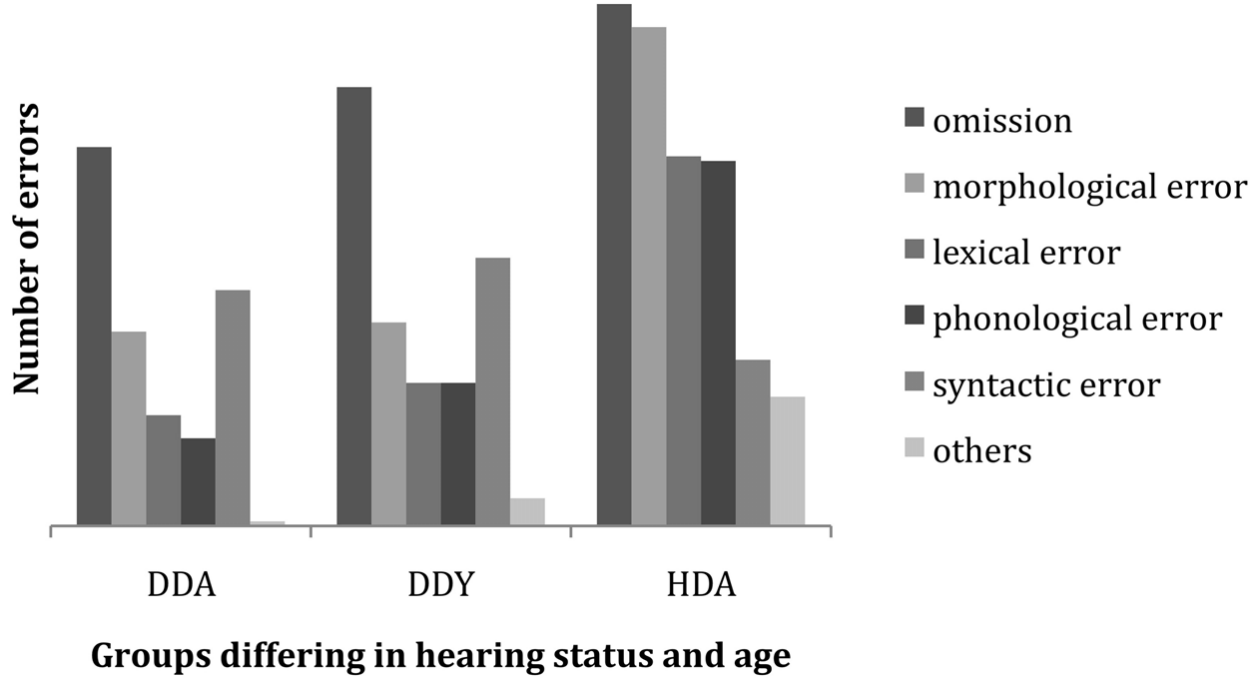
**Incidence and relative proportion of error types by hearing status and age**.

Our next step is to examine error patterns in the reproduction responses of signers as a function of their hearing status and age. Errors in signed responses are categorized by type and the proportion of each error type across the three groups in the subject pool is tallied. Figure 4 shows the striking distinction in error type distributions across the three groups. Between the HDA group and the other two groups, there is a contrast in the most prevalent types of error, in that these hearing signers make more morphological, lexical and phonological errors than the two groups of deaf signers, whereas omissions and syntactic errors are comparable across all three groups. This seems to indicate distinctive differences in the respective groups' strategies for performing the task.

A multiple analysis of variance (MANOVA) was conducted to determine if there were differences in the frequency of error types (Omit, Morphology, Lexical, Phonological, Syntax) between three groups of native ASL participants (Deaf adults, Deaf youth, Hearing adults). Significant group differences were found for Morphological errors [*F*_(2, 72)_ = 28.11, *p* < 0.001, eta squared = 0.44], Lexical errors [*F*_(2, 72)_ = 12.70, *p* < 0.001, Partial eta squared = 0.26], and Phonological errors [*F*_(2, 72)_ = 11.11, *p* < 0.001, Partial eta squared = 0.24]. There were no significant group differences for Omission or Syntactic errors. *Post-hoc* analyses with Bonferroni corrections revealed that Deaf adults and youth made fewer Morphological, Lexical and Phonological errors than Hearing adults. All were at *p* < 0.001 with the exception that the level of significance between Deaf youth and Hearing adults for Phonological errors was at *p* < 0.01. Deaf adults and youth had the same pattern of occurrence of errors across error types.

However, this is not the whole story. To understand the role and nature of cognitive mechanisms across levels of fluency, we re-grouped the subjects into high, moderate and low fluency groups. Then we investigated the strategies for the task within and across these groups as revealed by an in-depth error analysis. The results of this analysis are set out in the sections below.

#### Error types as a function of fluency

The 75 subjects were ranked based on their ASL-SRT performance as judged by the accuracy scores, and subjects with the top 25 highest correct reproductions were grouped as High (10 DDAs, 13 DDYs, 2 HDAs), the middle 25 as Moderate (11 DDAs, 6 DDYs, 8 HDAs), and the bottom 25 as Low (4 DDAs, 6 DDYs, 15 HDAs). The purpose of this re-grouping was to examine and describe the type of errors made by individuals of different levels of fluency across hearing status and age. In the figure below, we compare and contrast the error patterns of subjects who performed in the High (20–15 correct reproductions), Moderate (15–12 correct reproductions), or Low (12–2 correct reproductions) range.

**Figure 5:**
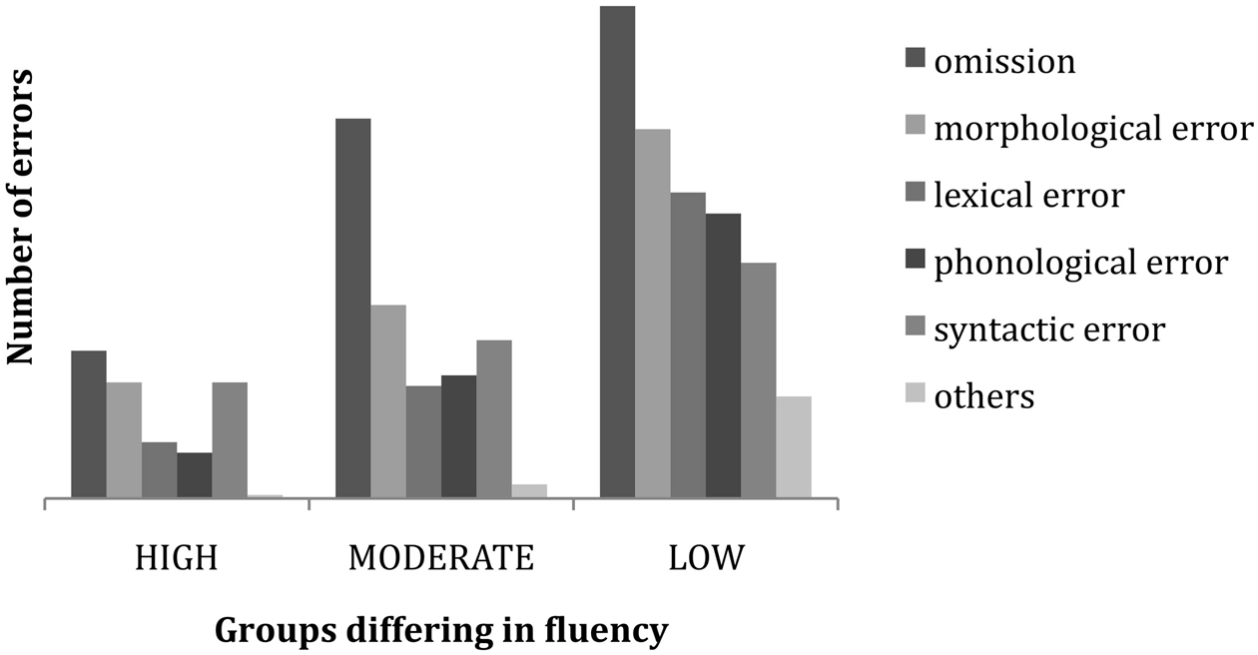
**Incidence and proportion of error type by fluency level**.

Figure 5 below shows how the relative proportion of error types interacts with subject fluency, producing differing proportions of error types for signers at the high, moderate and low levels of ASL fluency.

A multiple analysis of variance (MANOVA) was conducted to determine if there were differences in the error types (Omission, Morphological, Lexical, Phonological, Syntactic) as a function of sign fluency (High, Moderate, Low). Significant fluency differences were found for all error types: Omission errors, *F*_(2, 72)_ = 25.72, *p* < 0.001, Partial eta squared = 0.42; Morphological errors, *F*_(2, 72)_ = 19.79, *p* < 0.001, eta squared = 0.36; Syntactic errors, *F*_(2, 72)_ = 3.23, *p* < 0.01, eta squared = 0.08; Lexical errors, *F*_(2, 72)_ = 20.83, *p* < 0.001, Partial eta squared = 0.26; and Phonological errors, *F*_(2, 72)_ = 14.83, *p* < 0.001, Partial eta squared = 0.30.

*Post-hoc* analyses with Bonferroni corrections revealed that the Low fluency group made more Omission errors (*p* < 0.01) and Syntactic errors (*p* < 0.05) than the High fluency group. In addition, the Low fluency group made significantly more Phonological errors than both the Moderate (*p* < 0.01) and the High (*p* < 0.001) fluency groups. The Low and Moderate fluency groups made significantly more Morphological and Lexical errors (both *p* < 0.001) than the High fluency group but did not differ from each other.

### Differentiating the error patterns of fluency groups

Our qualitative investigation of errors reveals that the overall structure of reproductions by fluent native signers under extreme task demand, while not perfectly correct, is nonetheless consistently well-formed, regardless of whether the signer is deaf or hearing. In contrast, among less fluent signers there is an increase in ungrammatical responses, with some target words omitted or replaced with unintelligible forms. This trend increases as the length and morpho-syntactic complexity of the sentences increase.

Even the first half of the ASL-SRT, made up of 10 sentences averaging four words long, led to reproduction failures among semi-fluent subjects. Although there was no increased length in words across these sentences, respondents experienced increasing difficulty due to the increase in morphological complexity. This is due to the fact that there are two types of stimulus items in the first half of the ASL-SRT. While all consist of a single clause, the first five test sentences contain only bare, uninflected lexemes, while the second five sentences have inflectional morphemes affixed to some of the lexical items. Some are aspectual inflections; others are nominal class markers (classifier morphemes). This difference produces an increase in the potential for bottleneck conditions through even the first half of the ASL-SRT.

The determination of what causes the complexity of reproduction in the second half of the ASL-SRT test is less straightforward. In the construction of the test, the items were ordered empirically, based on how accurately subjects responded. For these most complex 10 sentences, verbatim serial memory obviously becomes more challenging as the sentence becomes longer. While these items do involve multiple clauses, it is not clear what precise features of structural complexity contribute to the psycholinguistic complexity of the reproductions.

With this perspective on such cognitive bottlenecks, we would expect to see a boost in performance accuracy if the subject had an opportunity for rehearsal using a variety of heuristics to reproduce the individual word components or the overall sign sequence of a test sentence. There is, however, no opportunity for rehearsal in the ASL-SRT. The subject only has one chance to see the test sentence and must work through potential bottlenecks in processing to reproduce even very complex, lengthy sentences with only the resources on hand.

In a sense, our data support a straightforward insight: we would not expect semi-fluent signers to be able to use non-linear scaffolding in their working memory in the way that fluent signers can. Whatever kind of working memory semi-fluent subjects have will determine the quality of their performance on the ASL-SRT task. Within this group, potential misarticulations might be replaced with unintelligible forms whenever subjects are overwhelmed by the task. In this context, lexical misarticulation by fluent signers will often include replacement of particular features. In contrast, we would expect an unintelligible form by a non-signer to be articulated with no constraints on the linear segmentation or inflectional prosody. In the case of a relative lack of knowledge of sign language phonology and morphology, visuo-motoric imagery may be a useful *ad-hoc* solution when attempting to process and imitate a string of linguistic word forms presented in sign language.

In this sense, the ASL-SRT task is significantly different from list recall. In a list recall task, subjects' reproductions are limited by the number of words in the list and by classic phenomena such as primacy and recency. As in spoken languages, once an individual word has a particular function in relation to other words in the sentential sequence, this affects how this word is encoded and then reproduced. In short, then, a mechanism other than serial word-list memory is required to explain the error patterns which we find across our subjects.

Here, we may draw on recent research on sentence reproduction processing. A distinction in error types revealing automatic vs. effortful processing has been modeled by Ronnberg et al. (2008) as implicit vs. explicit processing. This psycholinguistic model of online processing and remembering highlights the efficiency of implicit processing. In related literature, Potter and Lombardi (1990) have suggested a model of verbatim recall by native, fluent language users which relies on conceptual storage and regeneration of language structure in memory. In their model, verbatim recall of sentences relies on recent lexical activation.

## Results of error analyses

Fluent Deaf and Hearing signers obtained higher reproduction accuracy scores and made different sorts of errors than weak signers. The reproductions of highly-fluent subjects among the DDA, DDY and HDA groups often differed from the stimulus in lexical ways, while retaining and faithfully repeating underlying aspectual and other sub-lexical morphology. In contrast, weaker signers committed a greater number of morphological errors and omissions. Another distinctive profile among weak signers is that the number of well-formed words they produce (no matter if correct or not) remains constant throughout the test segments (which increase in complexity), revealing a specific cognitive limitation where grammatical knowledge is lacking. Also, any adult or child signer could potentially exploit visual and motoric imitation to overcome lexical and morphological limits, and this can lead to a superficial illusion of comprehensible signing. However, while we see such misarticulations among native signers across the range of fluency, these vary in their degree of grammaticality, with some showing a high formal resemblance to the target and others clearly non-signs. In this sense, the data reveal that signers' error types group according to individuals' relative level of competence and knowledge of ASL grammar. Thus, we would claim that the structure of the subjects' responses projects the affordance and constraints of deep linguistic representations to differing degrees, and subjects resort to alternate processing strategies in the absence of such knowledge or under conditions of high task demand. We will lay out observational generalizations highlighting these points and then provide interpretation of the data in support of the theoretical models cited above.

### Generalization 1: tendency toward simplification for particular word classes

The ASL-SRT task requires the subject to reproduce peripheral details along with main propositions. Occasionally a subject will eliminate peripheral details, especially determiners and qualifiers, when reproducing a target sentence. For example, the DET class in the subject NP position throughout the test is a construction prone to error. This is consistent both within and across subjects.

For certain word types, there are also constraints on the types of replacement errors produced, which tend to stay within the target class. For example, replacements for determiners stay within the class of determiners. The test items involve two different types of DET, but common replacements for both are the more generic INDEX or the target DET item without its spatial agreement inflection (indicated by ‘i’). Item #2 contains THATi (THAT with a spatial agreement inflection); items #8 and #10 contain SELF+locus-i (self with a spatial agreement inflection). In all of these cases, the target item is replaced by a less-marked DET of the same kind. It is rare that a more highly-marked or inflected DET form replaces a less-marked or uninflected form.

It appears that the surrounding context of a particular word can trigger a process that results in omitting or replacing a word or particle morpheme. This happens more often in the second half of the ASL-SRT (Items #11–20), where there are more signs to be reproduced and greater linguistic complexity of the sentences. As we will see in other examples, it appears that the sentence structure and task create a possible chain of errors in which the subject mistakenly encodes (or fails to encode) a definite or specified NP construction, thus taking a wrong turn in the processing of the sentence. Incomplete interpretation of the sentence can create such missteps in processing regarding a particular noun argument or its relation to other noun arguments, and the interdependence of grammatical operations can then lead to additional errors in the sentence.

### Generalization 2: interdependence of morpho-syntactic errors

Other reproductions of items containing DETs show that the position of the DET/specifier may shift, the DET may be omitted, or the DET may be copied to the beginning or end of the determiner phrase or of the entire clause. This can be seen in the reproductions of Sentence #20, where the determiner ONE appears beside the adjective LITTLE and the noun GIRL: ONE LITTLE GIRL. Two subject responses are: Target: ONE LITTLE GIRL vs. Response: GIRL LITTLE ONE or GIRL LITTLE. In the first response, the word order deviation can be viewed as a pragmatic variant, since bracketing of a phrase by a repeated determiner is a common ASL device for focus or emphasis; and prenominal adjectives are more frequently displaced after the noun rather than to any other position in the sentence. Alternatively, perhaps the subject initially omitted DET and ADJ by mistake and then filled in the omitted material afterwards. But in either case, the displacement is constrained, with the DET omitted or displaced to a position after the clause.

Omission of DET occurs most often among the subjects we tested and thus appears to be a common response to serial memory limitations during the reproduction task. In contrast, omission or misplacement of the head noun GIRL is rare, presumably due to its syntactic salience and to the fact that the adjacent words ONE and LITTLE depend on its appearance. Overall there is a hierarchical relationship among these three words, with their role in the phrase determining the likelihood of their appearance and position in responses. These data support a constraint-based theory of reproduction performance. Other classes of words (modals, qualifiers or quantifiers) follow a similar pattern.

### Generalization 3: processing chokepoints

In our analysis of sentence responses, we also identified specific intra-sentential locations where errors were likely to occur across all groups. We call these locations chokepoints: sentence locations where processing bottlenecks occur, as indicated by a high frequency of reproduction errors at that point in the sentence. However, the type and extent of errors in and beyond this point in the sentence were likely to be quite varied. The type of error resulting from a particular chokepoint depends on two factors: (1) the general fluency of the signer, and (2) lexico-morpho-syntactic complexity of a particular word in a sentence. The latter factor can induce a series of bottlenecks for a particular sentence item. Beyond this slot in the sentence, additional error types and number tend to cluster for signers, suggesting a non-linear hierarchy of grammatical domains constraining reproduction in these challenging conditions. The effects on a particular word can come from its visual, semantic or syntactic resemblances with different words in the lexicon or from its long-distance grammatical relations with other words in the sentence.

These chokepoints are not limited to a single grammatical domain. Earlier we illustrated the errors occurring within the grammatical domain of Determiner, for sentence items #2, #8, #10, and #20. In contrast, the errors for Sentence Item #4 as shown in Table 4 occur at several chokepoints where time and number signs occur.

**Table 4:** **Frequency and type of error made for sentence item 4**.

**Rank**	**Group**	**MY**	**LAST**	**VACATION**	**SEVEN**	**YEAR**	**AGO**
**ONE ERROR**							
19	DDY				Insert AGO (syntactic)		
48	DDY				Replace w/3 (phonological)		
65	HDA		Replace w/AGO (lexical)				
71	HDA		Replace w/AGO (lexical)				
73	HDA				Merge 7-YEAR (morphological)		
**TWO ERRORS**							
38	HDA		Replace w/AGO (lexical)	Replace w/CLASS (lexical)			
68	DDY		Replace w/AGO (lexical)		Replace w/3 (phonological)		
**THREE ERRORS**							
55	HDA	Omit	Replace w/AGO (lexical)	Omit			
75	HDA		Omit	Omit		Omit	

The error distribution in Table 4 shows an overall pattern like that for the adjective LAST as one primary chokepoint in the sentence. This slot in the sentence shows a greater number of errors occurring in comparison to the other items in the sentence. In terms of the subjects' thinking, the second word LAST may be confused with AGO; or some subjects may have only the sign AGO for expressing the meaning LAST. Such errors validate the concept of a sentence framework in which sentence slots that occur in a common linguistic frame may allow a swapping of words with a similar function or “spread” of the same word to a slot with a similar function. In these cases, we see the phenomenon detailed in Potter and Lombardi (1990), where a given conceptual content triggers a particular syntactic frame to be regenerated without verbatim recall.

Another chokepoint in this sentence is the numeral sign SEVEN. Subjects may misperceive how many fingers are extended, often replacing 7 with the numeral 3. Nevertheless, the replacement is still a numeral, a fact which demonstrates the constraints that their grammatical knowledge places upon their errors. Also in connection with this slot in the sentence, some subjects overgeneralize an ASL rule which allows for incorporating a numeral handshape into the following sign AGO, generally up to the number 5. Spreading the handshape for the numeral 7 throughout the following sign, YEARS-AGO, violates this combinatorial constraint, yet at the same time reveals greater knowledge of ASL structure.

In response to this sentence and in contrast to the types of errors above, some subjects produced a series of unintelligible forms. In some cases they placed their hand on their shoulder, thus indicating they noticed the articulation of the AGO segment. However, their choice of handshape was wrong, resulting in a non-sign. One young native signer misarticulated this sign by placing his hand on the opposite shoulder.

### Generalization 4: co-dependence among grammatical operations revealed in a range of surface outcomes

We now turn to examples of error patterns illustrating the interdependence of multiple processing strategies in the various grammatical domains of ASL. The sample analyses of responses here reveal the interdependent relation between misarticulation, omission and displacement made by subjects around chokepoints within individual sentence items. In ASL, assimilation across morphemic segments can result in a complex non-concatenative form. However, the fluent signer may cognitively parse these as separate morphemes during encoding of the stimulus sentence. This grammatical knowledge may then result in specific sorts of errors. Moreover, the resulting omission of a word may impact the well-formedness of the overall sentence response. However, the option for omitting a word is constrained by the grammar.

As described earlier, our analyses reveal a correlation between the grammaticality of the overall response and the fluency of the subject. The more accurate the subject was in performing the whole task, the more likely an omission is to be triggered by a grammatical operation generating an acceptable alternate sentence form. Sentence Item 6 is an example with a morpho-syntactic condition, which may trigger such top-down processing errors. In the target sentence, the negator NOT is separate from the verb LIKE. Subjects often merge NOT LIKE, preferring the alternate ASL contracted form. Some even produce double negation, in which they produce both the sequential NOT LIKE and the contracted form in the same sentence, a violation of the rule of negation in ASL.

One explanation of such errors is the independence of the negator in the modal domain vs. in its bound form in the verb-internal domain. A subject can fail to coordinate the two domains when functioning under bottleneck-inducing conditions. Furthermore the negative contracted verb may be encoded as a single lexeme and thus reproduced independently of other linguistic forms. This can lead to the redundant outcome.

At other times, the range of morphosyntactic commission choices mirrors the range of possible word replacements although certain replacements may be triggered by semantic and syntactic motivating factors, either within the local phrase structure or across multiple phrases. In contrast to the chokepoints in Sentence #4, which involve the grammatical domains of time and number, the errors in Sentence #7 as shown in Table 5 involve chokepoints which relate to linking/copular verbs and size-and-shape specifying classifier predicates.

**Table 5:** **Frequency and type of error made for sentence item 7**.

**Rank**	**Group**	**SUNDAY**	**NEWSPAPER**	**TEND**	**CL: thickness-on-surface**
**ONE ERROR**					
42	DDA				Replace primary CL (morphological)
48	HDA				Replace second CL (morphological)
52	DDA			Replace w/SENSITIVE (lexical)	
56	DDY	Omit			
59	HDA				Misarticulate w/CHURCH (phonological)
61	DDY				Replace second CL (morphological)
62	DDA			Omit	
66	HDA			Replace w/SOMETHING (lexical)	
**TWO ERRORS**					
58	HDA		Replace w/PAPER (lexical)		Replace primary CL (morphological)
69	HDA			Omit	Omit
70	HDA				Replace w/CHURCH (lexical)
71	HDA			Omit	Omit second CL (morphological)
72	HDA			Omit	Omit
74	HDA			Replace w/SENSITIVE (lexical)	Replace w/CHURCH (lexical)
75	HDA			Replace w/LIKE (lexical)	Replace w/CHURCH (lexical)
57	HDA			Replace w/SENSITIVE (lexical)	Replace primary CL (morphological) omit second CL (morphological)
68	DDY	Replace w/SATURDAY (lexical)		Replace w/NOW (lexical)	Misarticulate w/CHURCH (phonological)
**THREE ERRORS**					
73	HDA			Insert INDEX (syntactic) replace w/PREFER (lexical)	Omit second CL (morphological)

Such output may still show effects seen in STM, such as primacy or recency effects. Also among semi-fluent signers, we have found a larger proportion of visuo-motorically driven formation along with some linguistic fragments from ASL phonology. This shows that even a weak exposure and fluency level in ASL results in performance constraints of a grammatical nature rather than pure visual perception or imagery.

### Generalization 5: revealing relative grammatical proficiency via a left-to-right chain of errors

The rigorously-controlled ASL-SRT protocol helps to reveal behavioral patterns in the manual-visual modality among those who have had minimal opportunity for learning or experience to acquire genuine and complete linguistic encoding. Error trends among semi-fluent subjects suggest a kind of scaffolding mechanism that relies more on episodic memory, a type of memory that encodes experiences that are rich with temporal, visuo-spatial, and emotional information. As a result, when they respond to complex test items in the second half of the ASL-SRT, they often reproduce only 3–4 actual words and resort to unintelligible formation or omission for the rest of the items in the target sentence.

Even so, this behavior is still constrained by grammar. Some semi-fluent signers produced unintelligible attempts or omission of less familiar words throughout the sentence and reproduced familiar words accurately while maintaining the overall word order. Other subjects started recalling the sequence of familiar words in a row, as if they were maintaining the order of their appearance in the test stimulus. For the remainder of the sentence, they ended their response with unintelligible forms.

**Figure 6:**
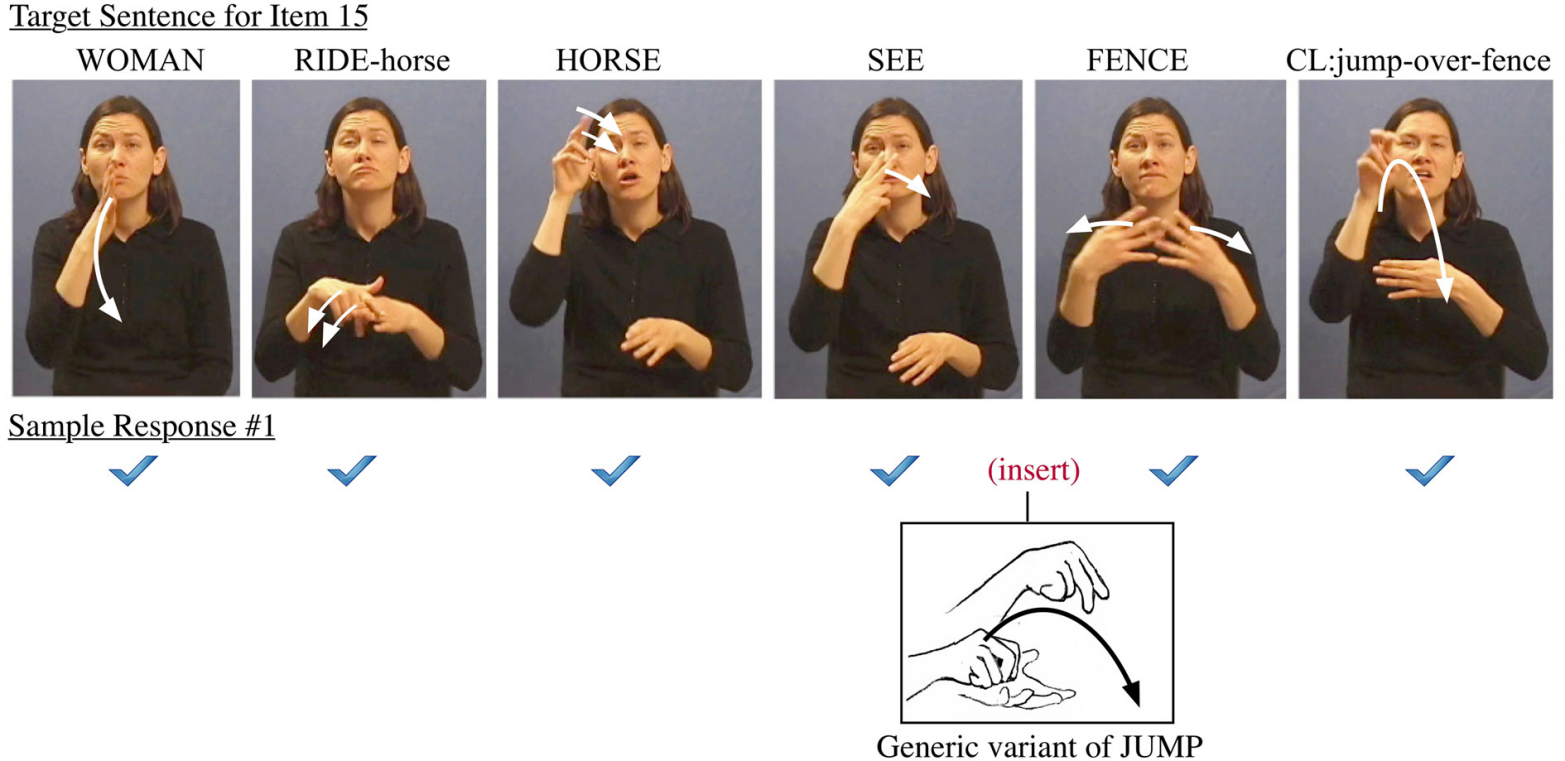
**Example of error made for sentence item 15**.

**Figure 7:**
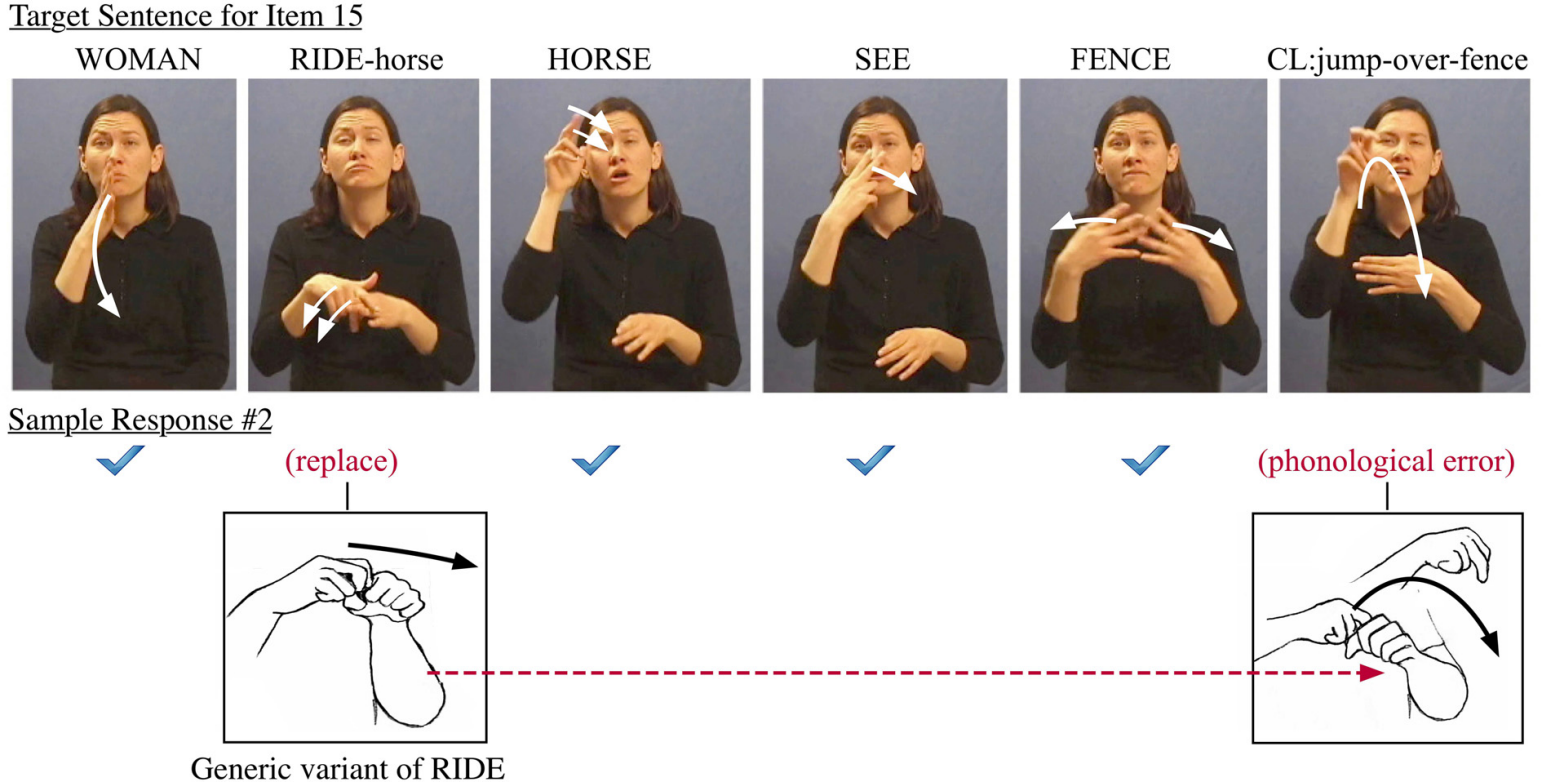
**Another example of error made for sentence item 15**.

**Figure 8:**
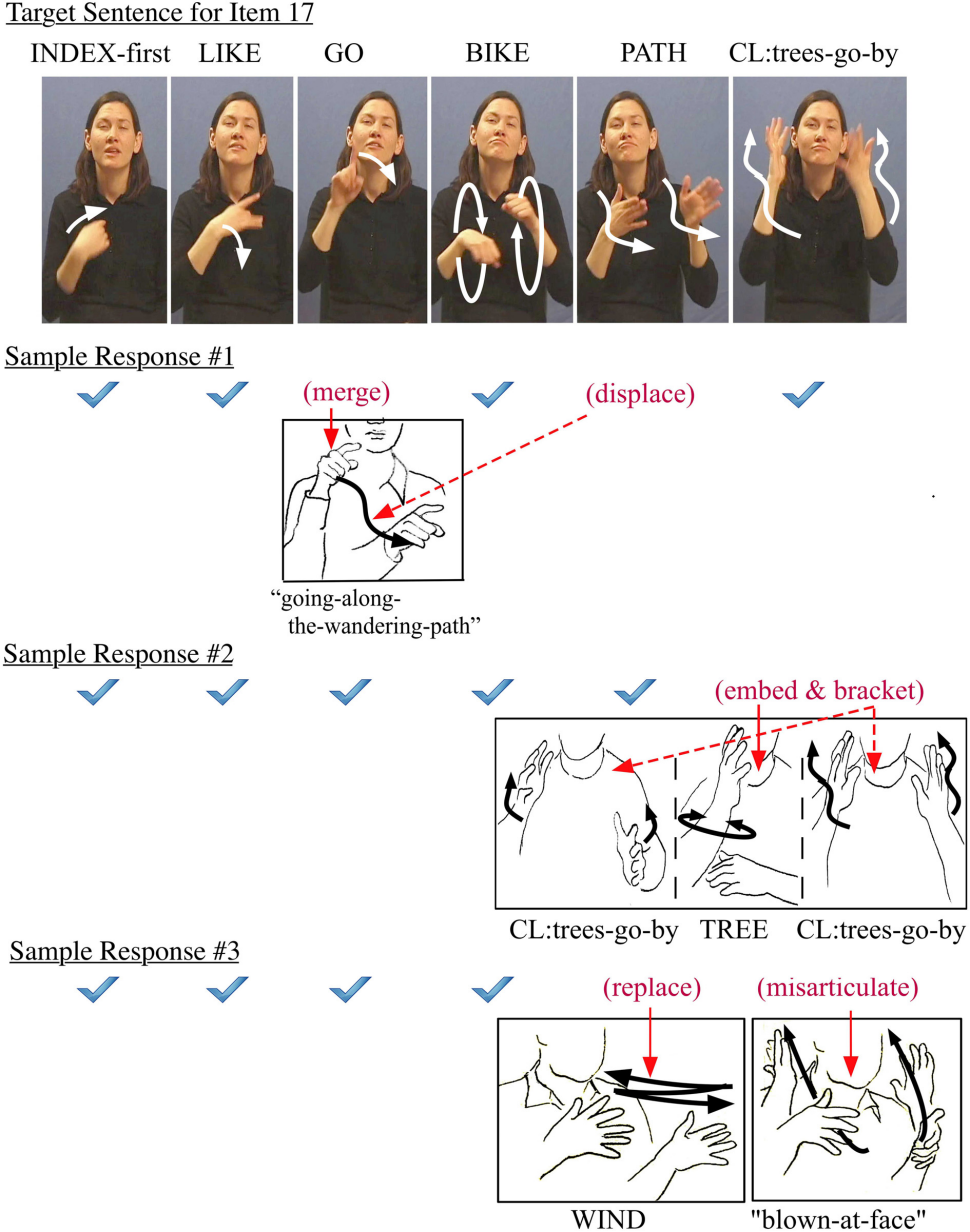
**Sample errors in responses to Item 17**.

It is essential to note that in all but the least-fluent signers, alternative options for sentence reproduction are constrained by grammatical boundaries for binding linguistic elements. The distance for displacement of a given word in a sentence, for example, is the result of a series of serial and parallel processing decisions. Such a “chain-reaction” phenomenon for reproduction is constrained by clause-internal restructuring as well as by the extent of the bottleneck and the increasingly severe types of errors it induces, such as the descent from local misarticulation into phrasal unintelligibility. Such interfaces are more complex in the test items involving multiple clauses. The error examples in Figures 6–**8** below are extracted from responses which are typical across all but the least-fluent signing subjects. The errors can show up in a variety of sentence response contexts, from a single isolated error to a series of related errors triggered by choices in sentence recomposition. As an example, the errors among adjacent words in Sentence Item #15, pictured in Figure 6, reveal several kinds of cascading interactions among the multiple operations for constructing poly-componential predicates in ASL.

In this response error, the signer bracketed the noun FENCE with the verb JUMP, once in a plain form and once with a locative form of the verb. In this case, the subject was able to merge the nominal class marker into the last verb as well, thus apparently introducing a serial verb construction (Fischer and Janis, 1990; Supalla, 1990). In other examples, the last verb is missing.

When adjacent words are merged in prosodic assimilation, we might assume the non-linear coalescence will reduce cognitive load, thus helping with the on-line processing of the sentence (Liddell and Johnson, 1989; Brentari, 1998; Sandler and Lillo-Martin, 2006). We often see such natural spreading of, for example, Weak Hand features to adjacent words. This is seen, however, only in specific morpho-syntactic contexts for fluent signers. For example, Sentence Item #15 has the prosodic scope of the spread Weak Hand feature extending from the verb RIDE to two subsequent words HORSE and SEE (see how the weak hand is maintained in the second and third photo in Figure 6).

Fluent signers do not extend such prosodic assimilation across phrasal boundaries into the sequence FENCE JUMP which involves poly-morphemic classifier constructions. In contrast, less-fluent signers do often assimilate in violation of the boundaries of words. This phonological assimilation can contribute to cascading errors, where less fluent signers may carry over the Weak Hand feature from RIDE to the last verb JUMP (see Figure 7). This correlates with their failure to merge the nominal class marker of FENCE into the last verb, since the Weak Hand is already occupied by the spreading Weak Hand feature from the earlier sign, as seen in the error below.

### Generalization 6: revealing grammatically constrained commissions

If comprehensible articulation is achieved, there is still a gradient of accuracy in word reproduction. Each error is either phonologically or syntactically constrained by available options. In other words, the signer selects specific linguistic properties for matching the target form. The choice of alternate features is likely to be formally constrained by the phonology when the subject making this sort of error is a native signer with adequate fluency. If sufficiently varying features make it clear that a different lexical representation is involved, then the error is identified as a lexical commission (i.e., word replacement for RIDE-horse with a generic variant of RIDE). Furthermore, in accordance with the syntactic operation triggering this feature spreading, the woman must be considered as the subject of the verb JUMP (and hence as the subject of the generic RIDE variant). The agrammatical response (“jump out of conveyance and leap into the air”) can be viewed as a phonological error, which is a consequence of the interpretation of the first verb in the target sentence.

From these sorts of errors, we see that success in reproducing Item #15 requires clausal scope for cognitive planning to preserve noun and verb relations. Moreover, Sentence #15 has an additional challenge, as the same hand configuration appears in several verbs throughout the sentence, with each use referring to a different noun argument. Such similarity in hand configuration can mislead some signers about noun relations. Evidence for this occurs in the errors of subjects who constructed responses in which the horse was the subject of the entire clause. Other subjects misunderstood the sentence in a different way, using the first subject WOMAN as the agent for jumping over the fence. Such syntactic errors are clearly grammatically constrained.

In the reproduction of sentence #17 on the ASL-SRT, there was a wide diversity in sequences of locomotion and path predicates. Figure 8 illustrates three sample responses to Item 17. The first example has the pointer morpheme merged with the path morpheme displaced from a subsequent word, leading to a morphological commission error in the outcome.

Wherever the original meaning was maintained while the target form was replaced, a particular response could be treated as acceptable for ASL (though not correct in the context of the ASL-SRT), as in the second example. As with other constrained error examples, the deep structure is the same while the surface structure reflects a different output as a result of an alternate combination of multiple target morphemes. Here a separate nominal lexeme TREE was embedded into the complex predicate TREES-GO-BY, resulting in a double bracketing of the predicate.

The third response example illustrates how visual cues may affect the subject's encoding of complex sentence stimuli. The lexical replacement WIND, and the subsequent need to insert a different sign following it, is a “chain reaction” effect and an unacceptable error. If we compare the three error examples, the first and second are acceptable (though not accurate), but the third is different, since the subject apparently reconstructs a structure from a partial short-term memory of the original stimulus. Here a series of deviating nominal and verbal morphemes were put together, resulting in a meaningful and grammatical phrase. However, this outcome was not a rephrase of the target sentence. In establishing categories of “good” vs. “bad” errors, we suggest that, for each word in a sentence item, there was a range of possible grammatical and agrammatical deviations from accurate reproduction.

## Discussion, conclusions, and implications

In our analyses thus far, we have discovered that overall accuracy on the ASL-SRT can be predicted by the hearing status and age of the signer. However, the best predictor of error types is the overall fluency of the signer. That is, fluent deaf and hearing signers differed from less fluent signers in the proportion of differing error types. Figure 5, left, represents the pattern of error types and their frequencies made by signers achieving scores in the top third of all signers on successful reproductions. At times, these data show some likelihood of either omitting a word or producing a grammatical alternate form. But overall these subjects maintain a high level of accuracy in sentence reproduction. The main distinction between those representing the middle third (moderately-fluent signers) and the least-fluent third is the choice of strategy for processing and performing our increasingly complex reproduction task. While these two groups generate similar numbers of errors, the moderately-fluent signers seem simply to amplify the error trends of more fluent signers. That is, they are more likely to omit a word or create a grammatical error involving either a morphological or syntactic alternation.

This supports the Potter and Lombardi (1990) claim that verbatim recall is due to recently activated lexical items coalescing into a coherent and rich conceptual trace for the sentence. In weaker subjects, there may not be a mental representation of particular lexical items, preventing verbatim recall of the normal type. Less fluent signers are instead more likely to misarticulate or replace words. The basic profile of lexical errors in ASL-SRT performance across fluency levels and sentence complexity reflects both lexical error commission and unintelligible misarticulations, with the latter increasing as fluency declines. Among weak signers the distributional pattern of lexical omissions, commissions and displacements is least predictable. These subjects are more likely to produce unintelligible forms, as if they are attempting to match the target form through visual-motoric imitation with no idea of what the word means. The criterion we used to distinguish an incorrect lexical item from an unintelligible form is the recognizability to the rater of the lexical root on which the misarticulation is applied. Careful investigation of the bar graphs in Figures 4, 5 reveals an increase in unintelligible forms (labeled as “other type of error”) as fluency decreases and an increase in alternate sign forms (often categorized as morphological or syntactic error) as fluency increases among native signers.

In this analysis, for highly fluent signers there is no random noise in the data, but instead a strongly constrained performance. What this may indicate is that once a subject achieves complete fluency, rapid processing and deep-structure grammatical/semantic coding is available as a top-down scaffolding route for working memory. Such cognitive bootstrapping from deep structure processing serves them well in the end, producing their top-end-skewed performance curve for the 20 sentence items in the ASL-SRT (See Figure 2). It seems that younger signers may not have yet achieved the far end of this curve, as seen by their drop in performance for the last few (most complex) test items.

In the ASL-SRT task, we hypothesize that the working memory performance is based on content-addressable memory structures, and not on ordered phonological representations like those used in the recall of random lists (Potter, 1990; Potter and Lombardi, 1990). Thus, the type of order information necessary during sentence reproduction processing is considered to be different from the slow, temporal order processing that mediates list recall (McElree et al., 2003; Lewis et al., 2006). This process is akin to the implicit processing incorporated in the Ease of Language Understanding (ELU) model of working memory by Ronnberg et al. (2008) and the conceptual regeneration process outlined in Potter and Lombardi (1990). The importance of conceptual representations for STM of scenes and sentences has been established in these works and in Haarmann et al. (2003).

In this sense, highly fluent signers' inaccurate responses can be partially attributed to paraphrase guided by deep structure processing, leading them to produce a cascade of lexical and morphosyntactic changes when, for example, the choice of an equivalent lexical item leads to an additional difference in the order of signs. This structured type of grammatical variation requires an architecture for coordinating multiple layers of linguistic processing for sentence decoding and recomposition. This likely involves the interaction of clause, phrase, and word levels, with the integration of features from different tiers of information orchestrated by an overarching representation of the meaning and structure of the sentence. In other words, the conventional model of serial processing for non-sentence material is here replaced by a hierarchical model with parallel processing capabilities, a top-down scaffolding mechanism that assists sentence reproduction. For subjects at different levels of fluency, there appear to be some important psycholinguistic differences:

1. For subjects with a low level of fluency, the encoding bias is toward a visuo-spatial strategy in which the surface physiological features of the hand configuration, handshape and hand movement trajectory are copied from the target. This often results in an unintelligible response, with no recognizable signs or grammatical features.
  At other times, the response may be more grammatical, but it will often involve multiple errors, each of a different type, independent of the others. Some signs may be omitted while others are misarticulated.
2. For moderately-fluent subjects, the bias is toward a linear strategy where individual signs and their syntagmatic positions are recognized and stored. The responses feature more accurate reproduction of the signs and their temporal sequencing in the sentence. Internal morphology is often deleted, and specific individual signs may be semantically replaced and/or phonologically misarticulated.
3. For highly-fluent subjects, the bias is toward a rapid processing of the semantic content of the sentence and a re-construction of the surface sentence composition guided by the deep structure grammar of ASL. Interestingly, this may put fluent native signers at a disadvantage in exact reproduction tasks. This often results in non-exact reproduction, as alternate grammatical forms for the stored meaning can be chosen and a shift in one grammatical element can cause cascading changes (scored as errors in an exact reproduction task) elsewhere in the sentence. This tendency increases with item difficulty. As a consequence, morpho-syntactic combinatory constraints often cause commission errors in the response. In this sense, while the test can function as a screening instrument for overall proficiency levels, its full value as a diagnostic instrument is realized with additional error analysis of responses.

In differentiating unintelligible articulation and constraint-based deviations at particular points of high task pressure, or “bottlenecks,” it is likely that the signing of an unintelligible response reflects a certain limit in working memory capacity, where the misarticulated form corresponds to the collapse of linguistic encoding. In contrast, more fluent signers may simply display errors of lexical commission, reproducing alternate morpho-syntactic configurations because they have been able to process sentences more deeply and rephrase the words to sustain the sentence meaning. Table 6 lays out our hypotheses about these on-line processing heuristics.

**Table 6:** **Modeling the correlation of error type to the layering of grammar**.

**Type of processing**	**Type of error**	**Domain of grammar**
Top-down	Syntactic re-phrase	Deep-structure and semantics
	Morpho-syntactic alternation	Syntactic inflection
	Syntactic displacement or reversal	Word order
	Morphological omission or alternation	Lexical inflection
	Lexical omission or commission	Lexical formation
	Lexical misarticulation or	Sub-lexical encoding
Bottom-up	unintelligible form	

The performance generalizations articulated above portray a psychological representation of this model. The escalating demands of the reproduction task result in clusters of various types of errors, which are useful for teasing out processing at the interface between the layers of processing and specific grammatical domains. In turn, this model accounts for how the cognitive system executes heuristic operations across domains and levels in both a serial and parallel fashion, thus making it possible to explain clusters of multiple errors in the ASL-SRT task.

The generalizations outlined above are consistent with several current models of general language processing. First, we see clear evidence for the model put forth in Potter and Lombardi (1990) of regeneration of conceptual content in accordance with grammatical constraints and prompted by recent lexical activations for verbatim recall. In highly fluent signers, we see cascading interactions among the multiple operations for constructing poly-componential predicates in ASL. In contrast, semi-fluent signers exhibit isolated error patterns when multiple errors in a single item are seen, indicating a lack of adequate conceptual understanding to create grammatical regeneration. Such errors also provide support for the Ronnberg et al. (2008) model of effortful “explicit” processing of individual lexical items, without the time to build the entire sentence through this process. Second, the retention of morphological concepts across sentence items in fluent signers indicates sentence comprehension and the formation of a sentence composition plan for a response, with working memory making use of the grammatical architecture to link morphemic constituents.

The inclusion of subject groups who vary in fluency levels has added rich data to the testing of such models of language processing. The intuitive distinction between “good” and “bad” errors reflects a sense of different types of cognitive organization across fluency levels. The coordination of individual linguistic operations to accomplish the reproduction task suffers as fluency decreases and task difficulty increases. For this analysis, we have posited three processing strategies in use by signers: top-down linguistic analysis, linear processing at the individual sign level, and visual-motoric “copying” of the stimulus. Each of these strategies points to a particular interaction between signer fluency and cognitive skills in accomplishing the reproduction task. We can imagine a hypothetical efficiency trajectory for each scaffolding strategy in sentence reproduction throughout the ASL-SRT task. Each strategy will peak at a particular point in the increasing complexity of potential bottleneck-inducing stimuli. In order to achieve further proficiency, a signer would need to switch from “episodic” to “linear” and finally to a “non-linear” type of scaffolding. Each of the strategies outlined above fits well within the models mentioned. These three strategies are: first, a strategy of visuo-motor episodic mimicry among semi-fluent signers; second, an explicit lexico-syntactic processing strategy where serial order is maintained; and third, a faithful top-down re-generation of sentence composition.

In episodic mimicry, we see an attempt to process language without a foundation for either explicit processing or conceptual regeneration. In the lexico-syntactic processing heuristic we see access to recent lexical activation without full or timely conceptual processing skills. In the reproduction of fluent signers, we see a range of possible “chain of error” outcomes, which may deviate from the stimulus for complex sentences. This indicates the availability of linguistic scaffolding and parsing options during cognitive and linguistic encoding and production.

## Conclusions and implications

The ASL-SRT test paradigm, with its increasing complexity and bottleneck conditions inducing errors in reproduction, reveals distinctive cognitive strategies across signers varying in fluency while controlling for language background. The specific details of a signer's experience with ASL in the home can apparently create the conditions for a particular heuristic strategy to be employed as part of that individual's available scaffolding and approach in coping with a stimulus item. This points to a range of cognitive strategies in working memory for the visual-gestural mode, which then interact with formal constraints of grammar to support the top-down processing capacity that fluent native signers possess.

While the data in the present study were all collected from native signers, there are similarities between what we have found as error types in our less fluent signers and error types that were found by Mayberry and Fischer (1989) in their study of sentence shadowing by native and late learners of ASL. A number of investigators have shown that late learners of ASL typically achieve lower levels of ASL fluency, even after full immersion and many years of language use (Mayberry and Fischer, 1989; Newport, 1990; Mayberry, 2010). As discussed earlier in this paper, Mayberry and Fischer's (1989) shadowing results showed that native signers' errors were predominantly semantic: they correctly represented the meaning of the target sentences, though sometimes changing the structure as they shadowed. In contrast, late learners' errors were predominantly phonological. This pattern is strikingly similar to the tendency of highly fluent signers in the present study to retain the deep structure of target sentences, whereas less fluent signers made a variety of more superficial errors and changes. Unfortunately we cannot discern without further analysis whether the representational and processing strategies of late learners are precisely the same as those of less fluent native signers, but these similarities in error types suggest that this may be the case.

The fact that this cognitive approach encompasses both the spoken and signed processing of language is noteworthy. Errors in the sentence reproduction task follow similar constraints as errors in natural language production. For example, lexical commissions usually respect word category, and misarticulations are constrained to possible word formation. Furthermore, this kind of data analysis has proven essential in our design of an efficient tutorial for increasing ASL-SRT raters' metalinguistic skills in detecting and categorizing response behavior.

The ASL-SRT holds promise as a research tool for the investigation of sign language processing across a variety of populations. In addition, the test can be applied to the screening, detection, and diagnosis of language behavior related to second language learning, language transfer and L1 intrusion, and age of acquisition issues, as well as for the detection and diagnosis of language impairment among native signers. Our future plans include presenting the ASL-SRT test to additional deaf native signers of varying ages, L2 hearing signers, late-learning congenitally deaf signers, and late-deafened signers as they progress through different levels of fluency in learning ASL. Such data will provide additional information on the heuristics used at different levels of fluency and knowledge of signed languages.

### Conflict of interest statement

The authors declare that the research was conducted in the absence of any commercial or financial relationships that could be construed as a potential conflict of interest.

## Footnotes

^1^A practical strategy in the absence of grammatical knowledge is revealed in the responses of a small pilot group of deaf college students who grew up without being exposed to sign language, learning ASL when entering college. Error analysis of these subjects' responses shows that they tend to use a strategy in which they attempt to copy the visual/motoric parameters of the signing stream. While this imitation may seem “correct” when the response resembles the stimulus, it often results in unintelligible signing. This sort of error has been noted in the literature. While Mayberry and Fischer (1989) did not include a non-signing “novice” group in their reported experiments, they do mention this sort of strategy among naive signers in a pilot group, calling it “hand waving” and differentiating it from the strategy and performance of even late-acquiring ASL signers.

^2^In Hauser et al. (2008) we described the development of a 39-item version of the ASL-SRT as well as its initial administration to populations with varied ASL backgrounds and skills. Initial administration of the test established the validity of the reproduction task as a tool for measuring fluency in a sign language, showing that the test does indeed discriminate among signers of varying ages and fluency. The current discussion focuses on a 20-item subset of the original 39 sentences. This subset of sentences comprises the second refined version of the ASL-SRT.

## References

[B1] Baddeley A. D. (1995). Working memory, in The Cognitive Neurosciences, ed Gazzaniga M. S. (Cambridge: MIT Press), 755–764

[B2] Baddeley A. D. Hitch G. J. (2007). Working Memory: past, present and future?, in The Cognitive Neuroscience of Working Memory, eds Osaka N. Logie R. H. D'esposito M. (New York, NY: Oxford University Press), 1–20

[B3] Bavelier D. Newport E. L. Hall M. L. Supalla T. Boutla M. (2006). Persistent differences in short-term memory span between sign and speech: implications for cross-linguistic comparisons. Psychol. Sci. 17, 1090–1092 10.1111/j.1467-9280.2006.01831.x 17201792

[B4] Boutla M. Supalla T. Newport E. L. Bavelier D. (2004). Short-term memory span: insights from sign language. Nat. Neurosci. 7, 997–1002 10.1038/nn1298 15311279 PMC2945821

[B5] Brentari D. (1998). A Prosodic Model of Sign Language Phonology. Cambridge, MA: MIT Press

[B6] Fischer S. D. Janis W. (1990). Verb sandwiches in ASL, in Current Trends in European Sign Language Research, eds. Prillwitz S. Vollhaber T. (Hamburg: Signum Press), 279–294

[B7] Fodor J. A. (1983). The Modularity of Mind. Cambridge, MA: Bradford

[B8] Haarmann H. J. Davelaar E. J. Usher M. (2003). Individual differences in semantic short-term memory capacity and reading comprehension. J. Mem. Lang. 48, 320–345 10.1016/S0749-596X(02)00506-5

[B9] Hammill D. D. Brown V. L. Larsen S. C. Wiederholt J. L. (1994). Test of Adolescent and Adult Language, 3rd Edn. Austin, TX: PRO-ED, Inc

[B10] Hauser P. C. Paludneviciene R. Supalla T. Bavelier D. (2008). American Sign Language-Sentence reproduction test: development and implications, in Sign Language: Spinning and Unraveling the Past, Present and Future, ed de Quadros R. M. (Petropolis: Editora Arara Azul), 160–172

[B11] Just M. A. Carpenter P. A. (1992). A capacity theory of comprehension: individual differences in working memory. Psychol. Rev. 99, 122–149 10.1037/0033-295X.99.1.122 1546114

[B12] Lewis R. L. Vasishth S. Van D. J. (2006). Computational principles of working memory in sentence comprehension. Trends Cogn. Sci. 10, 447–454 10.1016/j.tics.2006.08.007 16949330 PMC2239011

[B13] Liddell S. Johnson R. (1989). American Sign Language: the phonological base. Sign Lang. Stud. 64, 197–277 2087376

[B14] Mayberry R. (2010). Early language acquisition and adult language ability: what sign language reveals about the critical period for language, in The Oxford Handbook of Deaf Studies, Language, and Education, Vol. 2, eds Marschark M. Spencer P. (Oxford: Oxford University Press), 281–291

[B15] Mayberry R. Fischer S. (1989). Looking through phonological shape to lexical meaning: the bottleneck of non-native sign language processing. Mem. Cogn. 17, 740–754 10.3758/BF03202635 2811671

[B16] McElree B. Foraker S. Dyers L. (2003). Memory structures that subserve sentence comprehension. J. Mem. Lang. 48, 67–91 10.1016/S0749-596X(02)00515-621702779

[B17] Morford J. P. (2002). Why does exposure to language matter?, in The Evolution of Language Out of Pre-Language, eds Givon T. Malle B. (Philadelphia, PA: John Benjamins Publishing Company), 329–341

[B18] Newport E. L. (1990). Maturational constraints on language learning. Cogn. Sci. 14, 11–28 10.1207/s15516709cog1401_2

[B19] Newport E. L. Meier R. (1985). The acquisition of American Sign Language, in The Crosslinguistic Study of Language Acquisition, Vol. 1, The Data, ed Dan I. Slobin (Hillsdale, NJ: Lawrence Erlbaum Associates), 881–938

[B20] Potter M. C. (1990). Remembering, in An invitation to Cognitive Science, Vol. 3, eds Osherson D. Smith E. (Cambridge, MA: Bradford/MIT Press), 3–32

[B21] Potter M. C. Lombardi L. (1990). Regeneration in the short-term recall of sentences. J. Mem. Lang. 29, 633–654 10.1016/0749-596X(90)90042-X

[B22] Ronnberg J. Rudner M. Foo C. Lunner T. (2008). Cognition counts: a working memory system for ease of language understanding (ELU). Int. J. Audiol. 47(Suppl. 2), S171–S177 10.1080/14992020802301167 19012117

[B23] Sandler W. Lillo-Martin D. (2006). Sign Languages and Universals. Cambridge: Cambridge University Press

[B24] Supalla T. (1990). Serial verbs of motion in ASL, in Theoretical Issues in Sign Language Research, Vol. 1, eds Fischer S. D. Siple P. (Chicago: The University of Chicago Press), 127–152

